# Dynamics of apex and leaf development in barley as affected by *PPD-H1* alleles in two contrasting *PHYC* backgrounds under short or long photoperiod

**DOI:** 10.3389/fpls.2024.1398698

**Published:** 2024-09-03

**Authors:** Jorge D. Parrado, Roxana Savin, Gustavo A. Slafer

**Affiliations:** ^1^ Department of Agricultural and Forest Sciences and Engineering, University of Lleida-AGROTECNIO-CERCA Center, Lleida, Spain; ^2^ Catalonian Institution for Research and Advanced Studies (ICREA), Barcelona, Spain

**Keywords:** *Hordeum vulgare*, flowering, developmental phases, Phyllocron, heading

## Abstract

Barley development from seedling to flowering involves both external and internal changes, the latter requiring microscopic observation. Internal changes allow for the classification of preflowering development into three phases: vegetative, early reproductive, and late reproductive. Genetic and environmental factors influence the duration of these phases, impacting grain yield. Photoperiod-sensitivity genes *PPD-H1* play a major role in flowering time, affecting adaptation; however, the effect might also be direct (beyond affecting phenology). In this paper, we aimed to assess how *PPD-H1* alleles affect barley development, including the progression of growth phases, leaf emergence, tillering dynamics, and spikelet development. Two experiments (field and controlled conditions) were conducted with a factorial combination of (i) four near-isogenic lines (NILs) for *PPD-H1* alleles (*ppd-H1* or *Ppd-H1*) under two contrasting *PHYC* genetic backgrounds (*PhyC-l* and *PhyC-e*) and (ii) two photoperiod conditions (short and long days). As expected, longer photoperiods led to a shorter growth cycle. All subphases of time to flowering, final leaf number, and phyllochron were affected by photoperiod. The effects of *PPD-H1* on flowering time depended on the *PHYC* genetic backgrounds and photoperiod conditions. *PPD-H1* effects on flowering time were associated with leaf number and phyllochron; the interplay between leaf number and phyllochron affected mainly the late reproductive phase. We also found that although *PPD-H1* did not affect the phyllochron of the first six leaves, the phyllochron of leaves appearing later, when grown under a short photoperiod, was consistently increased in lines carrying the *ppd-H1* allele. Tillering dynamics exhibited variability, but *PPD-H1* did not affect the final spike number under a 24-h photoperiod.

## 1 Introduction

Barley development from seedling emergence to flowering encompasses changes that are both external, visible to the naked eye, and internal, requiring dissection of the meristematic apex and observation under the microscope. Internal changes are the basis for the partitioning of time to flowering into a sequence of three consecutive phases: (i) vegetative (from seedling emergence to the first double ridge^1^, mostly a leaf primordia initiation phase), (ii) early reproductive (from first double ridge to awn initiation, basically the spikelet initiation phase), and (iii) late reproductive (from awn initiation to flowering, when the survival of initiated spikelets takes place, resulting in the number of fertile florets) (Appleyard et al., 1982; Kirby and Appleyard, 1984; Sreenivasulu and Schnurbusch, 2012). The periodic determination of the number of spikelets initiated and the stage of floret development in each of them allows the determination of the dynamics of floret initiation and mortality, determining spike fertility, a major driver of yield in small grain cereals (Slafer et al., 2022; Serrago et al., 2023). External changes include the number of structures (number of leaves on the main shoot, number of tillers) that, when measured periodically along the season, allow determining the dynamics of both leaf appearance and tillering (Zadoks et al., 1974; Slafer and Rawson, 1994; González et al., 2002; Slafer et al., 2015). Both dynamics are relevant, the former because the time to flowering is strongly related to both the number of initiated leaves in the apex during the vegetative phase and their rate of appearance, and the dynamics of tillering and tiller mortality is relevant as they define the number of spikes, which is also a relevant component of barley yield (Miralles et al., 2021; Serrago et al., 2023).

The duration of preflowering phases, when major yield components are being formed in cereals (Slafer et al., 2023b), is controlled by genetic and environmental factors (Andrés and Coupland, 2012; Casal and Qüesta, 2018). Indeed, specific yield components are formed during distinct phases of plant development (Slafer and Rawson, 1994). Several studies reported phenotypic variability in the duration of preflowering phases among genotypes with similar flowering time (Appleyard et al., 1982; Kitchen and Rasmusson, 1983; Kernich et al., 1997; Whitechurch et al., 2007a, 2007b). Therefore, not only time to flowering is relevant but also the distribution of that time across its different subphases when affected by genetic or environmental factors.

Photoperiod sensitivity genes are critical for determining the time to flowering and adaptation in barley. Although there are two major photoperiod-sensitivity genes, *PPD-H1* is by far the most relevant and, therefore, the primary target for improving barley adaptation (Turner et al., 2005; Jones et al., 2008; Wang et al., 2010; He et al., 2019; Fernández-Calleja et al., 2021). Barley is a quantitative long-day plant that accelerates its development under long photoperiods (Boyd et al., 2003; Karsai et al., 2004). The allelic version of *PPD-H1* modifies the photoperiod sensitivity (i.e., the dominant allele, *Ppd-H1*, confers photoperiod sensitivity, while the recessive allele, *ppd-H1*, is known as the photoperiod-insensitive^2^ allele, even though it does also confer sensitivity, but noticeably less than *Ppd-H1*) (Laurie et al., 1994; Turner et al., 2005; Von Korff et al., 2010; Parrado et al., 2023; Slafer et al., 2023a). In fact, the effect of *PPD-H1* alleles on time to flowering in spring barley tends to be maximised at intermediate-long photoperiods (e.g., 12–16 h; Fernández-Calleja et al., 2021, and references therein), but minimised at extremely long photoperiods 21–24 h (Parrado et al., 2023).

Previous studies suggested a pleiotropic effect of the *PPD-H1* gene on yield components within the classical photoperiod range of 12 to 16 h (Von Korff et al., 2006; Wang et al., 2010; Borràs-Gelonch et al., 2012; Ponce-Molina et al., 2012; Pankin et al., 2014; Bustos-Korts et al., 2019; Wiegmann et al., 2019). Determining whether *Ppd-H1* has true pleiotropic effects (beyond those on time to flowering) is required to grow the plants with contrasting photoperiod sensitivity at a photoperiod in which they flower simultaneously. In a previous paper (Parrado et al., 2023), we showed that under extremely long days, *PPD-H1*-sensitive and *PPD-H1*-insensitive lines tend to flower simultaneously. Consequently, under these conditions, genetic effects not associated with the crop cycle could be studied. In this scenario, we attempted to synchronise the flowering time of all lines, regardless of their photoperiod sensitivity, by saturating the photoperiod response with 24-h daylength and then studying whether these genes affect developmental components independently of flowering time. To gain consistency of conclusions regarding the possible true pleiotropic effects of *PPD-H1* on yield components or to show relevant interactions conditioning such effect, it would be beneficial to test the effects of *PPD-H1* alleles under contrasting genetic and environmental backgrounds.

Another gene affecting flowering time in barley related to the perception of light is the red/far-red light photoreceptor phytochrome C (*PHYC*), which is closely linked to *VRN-H1* (Szucs et al., 2006; Nishida et al., 2013; Pankin et al., 2014). Under vernalised conditions, *VRN-H1* would not have an effect on time to flowering; when both linked genes are modified together, any effect on time to flowering would be driven by the *PHYC* late- and early-flowering alleles (*PhyC-l* and *PhyC-e*, respectively; Ochagavía et al., 2022).

The aim of this study was to assess the effects of *PPD-H1* alleles on the phasic, leaf, tiller and spikelet development of barley. To strengthen the robustness of conclusions reached, we compared near-isogenic lines with *Ppd-H1* and *ppd-H1* alleles combined with contrasting *PHYC* backgrounds and under contrasting photoperiod conditions (i.e., we quantified the effects of *PPD-H1* alleles against contrasting overall times to flowering given by genetic and environmental factors) in experiments under field and controlled conditions.

## 2 Materials and methods

### 2.1 Experimental conditions and treatments

Two experiments (field and controlled conditions) were conducted during the 2019–2020 growing season. Treatments in each of the experiments consisted of a factorial combination of (i) four near-isogenic lines (NILs) for *PPD-H1* alleles (*ppd-H1* or *Ppd-H1*) under two contrasting *PHYC* genetic backgrounds (*PhyC-l* and *PhyC-e*) and (ii) two photoperiod conditions (short and long days). NILs were produced at CSIRO (Canberra, Australia) after five cycles of backcrossing, using different donors of *VRN-H1/PHYC* and *PPD-H1* alleles into the facultative recurrent barley cultivar “WI4441” (Oliver et al., 2013).

The four genotypes were actually aimed to be isogenic for allelic constitution of *PPD-H1* and *VRN-H1*, but as the latter is closely linked to *PHYC* (Szucs et al., 2006; Nishida et al., 2013; Pankin et al., 2014), the NILs were actually *vrn-H1+PhyC-e* and *Vrn-H1+PhyC-l* (**Table 1**), as demonstrated by Ochagavía et al. (2022) who genotyped these NILs, finding that winter (*vrn-H1*) lines carried the *PhyC-e* allele and spring (*Vrn-H1*) lines the late allele (*PhyC-l*). Although this linkage prevents a clear separation of the effects *VRN-H1* and *PHYC* genes, in the experiments reported here, plants were vernalised (see below), and therefore there were no effects of *VRN-H1* on any developmental attribute. Thus, for simplicity, we considered herein these NILs as the combinations of the two allelic constitutions of *PPD-H1* and *PHYC* (**Table 1**). All lines had the dominant *Vrn-H2* and *Ppd-H2* alleles and haplotype II of *HvCEN* (Oliver et al., 2013; Ochagavía et al., 2022); i.e., all effects on developmental characteristics will be due to the action of *PPD-H1* alleles under the particular backgrounds of contrasting alleles of *PHYC* and contrasting photoperiods.

**Table 1:** Allelic constitution of barley NILs analysed in this study for *PPD-H1* and *VRN-H1* + *PHYC* genes.

Photoperiod sensitivity	*PPD-H1* allele	Earliness due to *PHYC*	*VRN-H1* + *PHYC*	Denomination in this study
Sensitive (Ps)	*Ppd-H1*	Early (Ea)	*vrn-H1 + PhyC-e*	PsEa
Insensitive (Pi)	*ppd-H1*			PiEa
Sensitive (Ps)	*Ppd-H1*	Late (La)	*Vrn-H1 + PhyC-l*	PsLa
Insensitive (Pi)	*ppd-H1*			PiLa

The field experiment (Exp1) was sown on 03 December 2019 in a facility with photoperiod control available at the campus of the University of Lleida, Spain (41°37′50″N, 0°35′27″E; altitude 180 m) in a fine loamy, mixed (calcareous), thermic soil classified as Typic Xerofluvent, according to the USDA taxonomy (Soil Survey Staff, 1999). Seeds of each material were distributed in strips of biodegradable paper, ensuring a uniform distance between plants within rows as well as a uniform seedling depth.

Plots were maintained throughout the whole cycle under either (i) natural conditions, with an average photoperiod from seedling emergence (SE) to flowering (Fw) of ca. 12 h (11.7 h ± 0.02 h), or (ii) a 24-h daylength, artificially extending the natural photoperiod with low-intensity (60 W) incandescent bulbs positioned on top of the designated plots. The radiation intensity was more than enough to produce the daylength signal, but increased radiation only negligibly (ca. ~ 3.6 μmol m^−2^ s^−1^ PAR at canopy level), below the light compensation point for barley (i.e., irradiance at which photosynthesis equals respiration and net photosynthesis is zero) normally around 10–15 μmol m^−2^ s^−1^ (Arenas-Corraliza et al., 2019; Chen et al., 2021), allowing plants to alter their developmental patterns but not affecting daily growth directly.

Exp1 was drip-irrigated when needed in order to avoid water stress. Weeds, diseases, and insects were controlled or prevented by spraying herbicides, fungicides, and insecticides at doses recommended by their manufacturers.

In the growth chamber experiment (Exp2), NILs were grown at the relatively low and constant temperature of 12°C (to expose plants to a temperature approaching the average temperature from SE to Fw more realistically than most controlled conditions growing temperate cereals that set growing temperatures at 18°C–25°C, accelerating development to minimise experimental duration). Indeed, the mean temperature from seedling emergence to flowering in the field experiment was 9.2°C. The two different temperature regimes in our experiments—lower average temperatures with natural daily and monthly variations in the field and slightly higher and constant temperatures in the growth chambers—along with other differences in the experimental setups could affect the strength of our conclusions. The conclusions will be more solid if the results are consistent across both experiments and weaker if the results are conflicting. The photoperiod treatments were 12 and 24 h; in the latter, only half of the lights were switched on during the duration of the day to compensate for the difference in daylength, so that in both conditions the daily radiation was the same (5.2 MJ m^−2^ day^−1^). In Exp2, seeds were germinated in 235 cm^3^ black plastic pots filled with 110 g of a soil mixture (70% w/w peat and 30% w/w organic amendment) freshly prepared before sowing. There was only one seedling per pot, and after being vernalised (see below), we transferred a set of 26 pots per NIL with seedlings at exactly the same stage (see below) to each of the two cabinets, previously configured for temperature and photoperiod conditions. Many of these plants were sampled for periodic dissections and intermediate determinations during the duration of the experiment, but at least three out of the 26 were left intact until flowering. Within each chamber, the pots were distributed randomly on trays and rotated at least twice weekly to avoid any possible positional effect within the chamber. Plants were irrigated daily, and each pot was fertilised with both macro- and micronutrients to avoid nutritional deficiencies.

**Figure 1:**
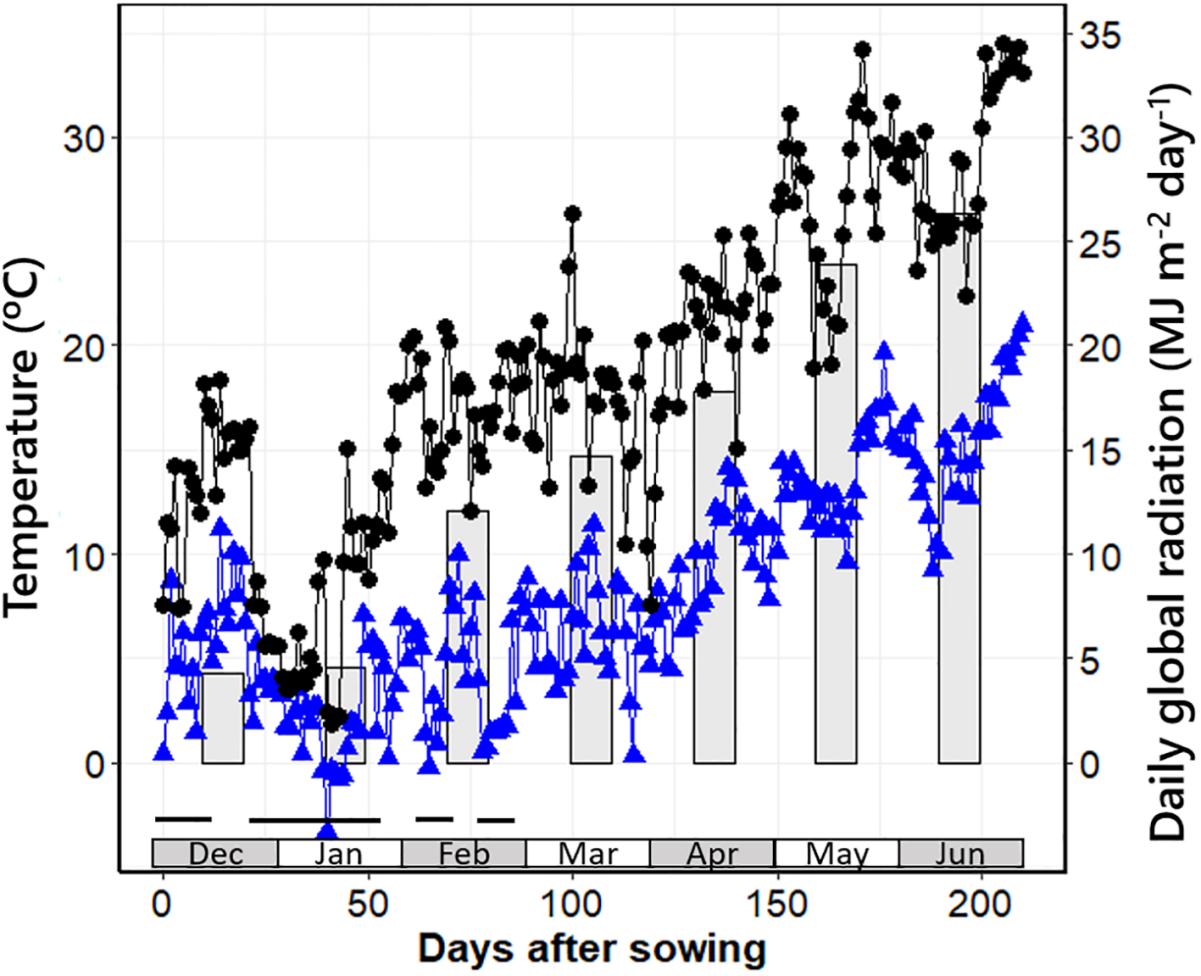
Minimum and maximum daily temperatures (triangles and circles, respectively) and daily global radiation averaged per month (bars) from sowing date to harvest of the latest plots in Exp1. The black horizontal bars at the bottom of the graph indicate periods with mean daily temperatures below 10°C.

In both experiments, all plants were vernalised; in Exp1, plants were naturally vernalised when exposed to winter (as they were sown in late fall). From sowing to the end of winter, seedlings were exposed to 41 fully vernalising days (days with mean temperatures with maximum effect on vernalisation, between 0°C and 8°C; Flood and Halloran, 1986; Brooking and Jamieson, 2002; **Figure 1**) plus 26 days with mean temperatures between 8°C and 10°C [that are also strongly vernalising temperatures, considering that vernalisation is produced when temperatures are up to 15°C; Brooking and Jamieson (2002)]. In Exp2, pots were exposed to vernalising temperature (4°C constant during the whole day) for 29 days in a cold room. Firstly, the pots were filled and sown at exactly the same depth with one seed per pot, but with 35% more pots than needed for the experiment (i.e., we sowed and included 70 pots of each individual NIL in the vernalisation pretreatment; in each of the two growth chambers prepared for the experiment, we transferred only 26 pots per NIL). This allowed us to discard not only the few pots in which seedlings did not emerge but also the tails of early- and late-emerging seedlings. As a result, when the experiment started and we transferred the pots from the vernalisation room to the growth chambers at 12 or 24 h photoperiod, all plants were extremely uniform (averaging 1.06 ± 0.02 emerged leaves).

After sowing the pots, before transferring them to the cool room for vernalisation, they were watered and left for 1 day at room temperature to trigger the germination process. Subsequently, all pots were transferred to a cool room. Finally, the 52 pots per NIL selected for having homogeneous seedlings were transferred to the growth chambers, and the experiment started (and for simplicity and using the same terms in both experiments, the date of starting the experiment was identified as “seedling emergence”, which, strictly talking, was slightly later in Exp2).

Treatments in Exp1 were arranged in a split-plot design, where the main plots, allocated to three complete blocks, were assigned to the photoperiod treatments, and the subplots, allocated randomly within the main plots, were assigned to the NILs. Subplots were 3.5 m in length and 1.2 m wide, with six rows (0.2 m apart) and a seedling rate of 200 plants m^−2^. In Exp2 within each cabinet, a set of 26 barley plants of each of the four NILs (i.e., 104 plants in each photoperiod condition) were arranged in a complete randomised design.

### 2.2 Measurements and analyses

The duration of both time from seedling emergence to flowering and of the phases composing it (i.e., from seedling emergence to awn initiation [SE-AI], from then to flag leaf [AI-FL], and from then to flowering [FL-Fw]) was expressed in thermal time, using the average temperature recorded at the site in Exp1 (Meteorological station from the Meteorological Service of the Government of Catalonia [Meteocat]) and the temperature of the chamber in Exp2, assuming a base temperature of 0°C, as standardly done (Kirby, 1988). The developmental stages determined (SE, AI, FL, and Fw) were in accordance with the Zadoks’ scale (Z09-10; Z31-33; Z39; Z55; Zadoks et al., 1974). However, for a more accurate determination, AI and Fw were determined, taking into account internal structures not normally visible to the naked eye. Awn initiation was determined microscopically when the tip of the lemma primordium started to grow and curve over the stamen primordia (~ W4.5). Flowering was determined as the time of pollination by regular microscopic dissection of the main spike and determining when it reached stage 10 on the Waddington et al. (1983) scale (i.e., when styles are curved outward with stigmatic branches widely spread and pollen grains visible on stigmatic hairs).

From SE to Fw, main stems were monitored once a week to determine the duration of different phenological phases [as delimited by stages determined externally by the scale of Zadoks et al. (1974) and internally by the scale of Waddington et al. (1983)]. In addition, three plants per experimental unit were randomly selected^3^ and tagged soon after SE, and the number of leaves that emerged on the main shoot was recorded twice a week following the scale developed by Haun (1973), while simultaneously the number of emerged and living tillers were determined.

From SE onward, representative plants of each NIL (three in each experimental unit of Exp1 and two in each chamber of Exp2) were sampled twice a week, and apical development was observed under the microscope after dissecting the main shoot apex. In addition, a detailed morphological analysis of spikelet and floret development of the main shoot spikes was carried out following the scale described by Waddington et al. (1983). The apices were dissected under a stereomicroscope Leica MZ 80 (Leica Microscopy System Ltd., Heerbrugg, Switzerland) equipped with a digital camera (model DFC420, Leica).

Phyllochron (i.e., the thermal time interval between the appearance of two successive leaves) was calculated as the reciprocal of the rate of leaf appearance (i.e., the slope of the relationship between the cumulative number of leaves on the main shoot and the thermal time). Whenever a linear model did not produce a random distribution of residuals, a bilinear model was fitted (with one phyllochron for the first leaves and another one for the last leaves) and, in these cases, considering the average phyllochron of all leaves as well as those for early- and late-appearing leaves.

Analysis of variance (ANOVA) was used to partition variation into effects of treatments and their interactions using the statistical software JMP^®^ Pro version 16.0 (SAS Institute Inc., Cary, NC, USA). Differences among means were compared using the least significant difference test (LSD, considered to be statistically significant if *p* < 0.05). To assess the degree of relationships between variables, linear regression analyses were performed. Polynomial regressions (Loess smooth line) were performed for the numbers of leaves, tillers, and floret dynamics, using an alpha of 0.75 and 95% confidence interval. Graphs were created in R using the package “ggplot2” (Wickham, 2016; R Core Team, 2020).

## 3 Results

### 3.1 Phenology

**Figure 2:**
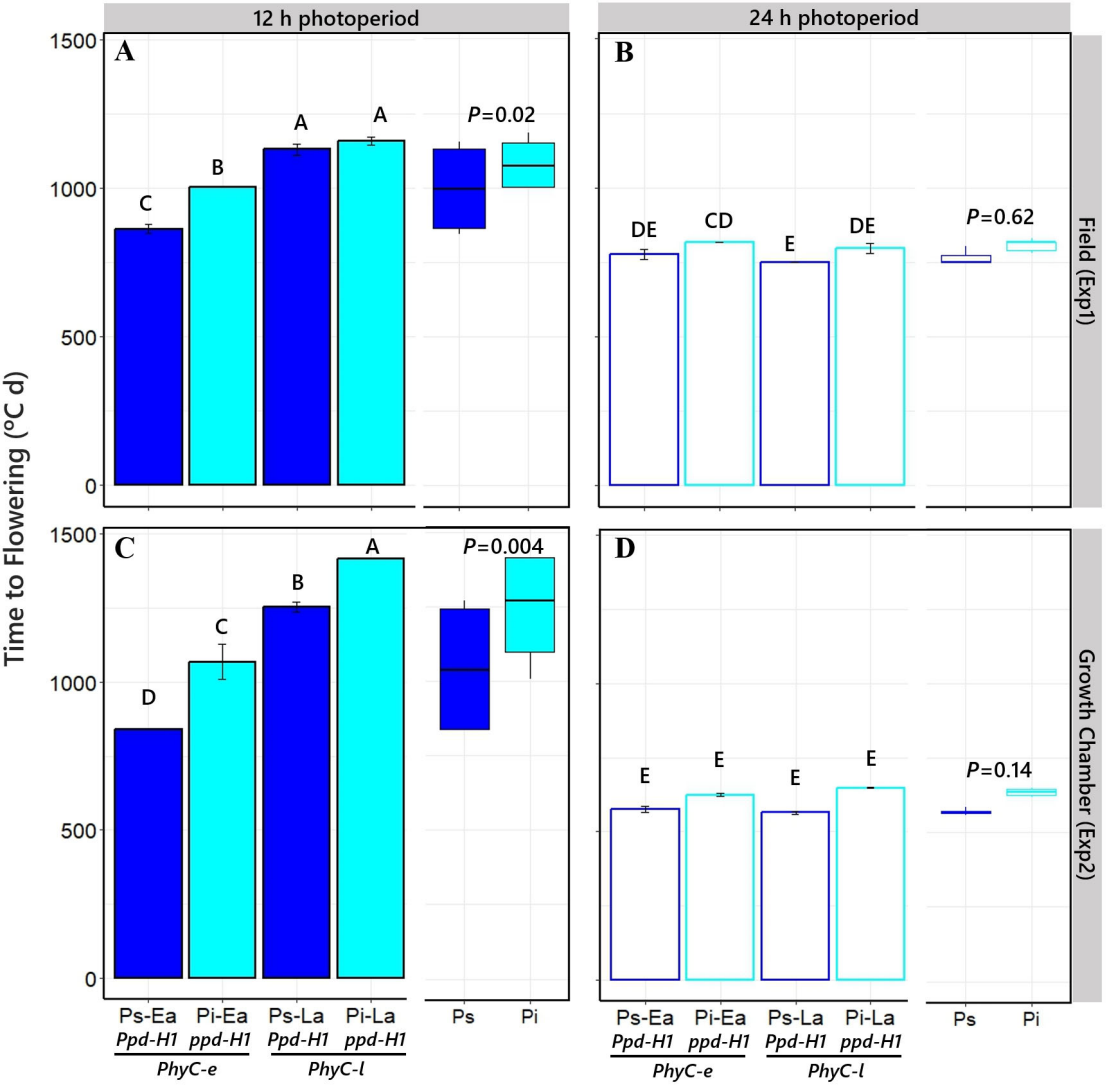
Time to flowering of the four different NILs grown under short [12 h; **(A, C)**] and long [24 h; **(B**, **D)**] photoperiods in field and growth chamber conditions (top and bottom, respectively). Different capital letters indicate significant differences (*p* < 0.05) between NILs with *Ppd-H1*-sensitive (Ps, dark blue bars) and *ppd-H1*-insensitive (Pi, light blue bars) alleles, combined with each of the two *PHYC* backgrounds within the left and right half of each panel, *PhyC-e*: early (Ea); PhyC-l: late (La). In the field experiment, a 12-h photoperiod corresponds to the average of the period from seedling emergence to flowering. Boxplots in each panel represent time to flowering for *Ppd-H1*-sensitive (dark blue) and *ppd-H1*-insensitive (light blue) alleles grouped across *PHYC* backgrounds, including the level of significance (*p*-value) of the difference between NILs with contrasting *PPD-H1* alleles within each photoperiod treatment.

As expected for a quantitative long-day plant, the overall duration of the cycle from SE to Fw was reduced when plants were grown under long days (*cf*. right and left panels in **Figure 2**). More relevantly, in the context of the aims of this study, the effect of *PPD-H1* gene on time to flowering in each of the contrasting *PHYC* genetic backgrounds depended on the photoperiod condition. There was an interaction between NILs and photoperiod on time to flowering: at 12 h photoperiod, Fw was delayed by the action of the *ppd-H1*-insensitive allele (although the effect was a nonsignificant trend when the *PhyC-l* allele was in the background in Exp1, the direct effect of *ppd-H1* was still significant when considered across the two *PHYC* backgrounds; see boxplots in **Figure 2A**), while under 24 h photoperiod, the *ppd-H1* allele did not significantly delay Fw (**Figures 2B, D**). The responses of time to Fw caused by *PPD-H1* across the two *PHYC* backgrounds were clearer under controlled conditions, but importantly, we observed the same effects in the field (*cf*. see boxplots in **Figures 2A, C**).

**Figure 3:**
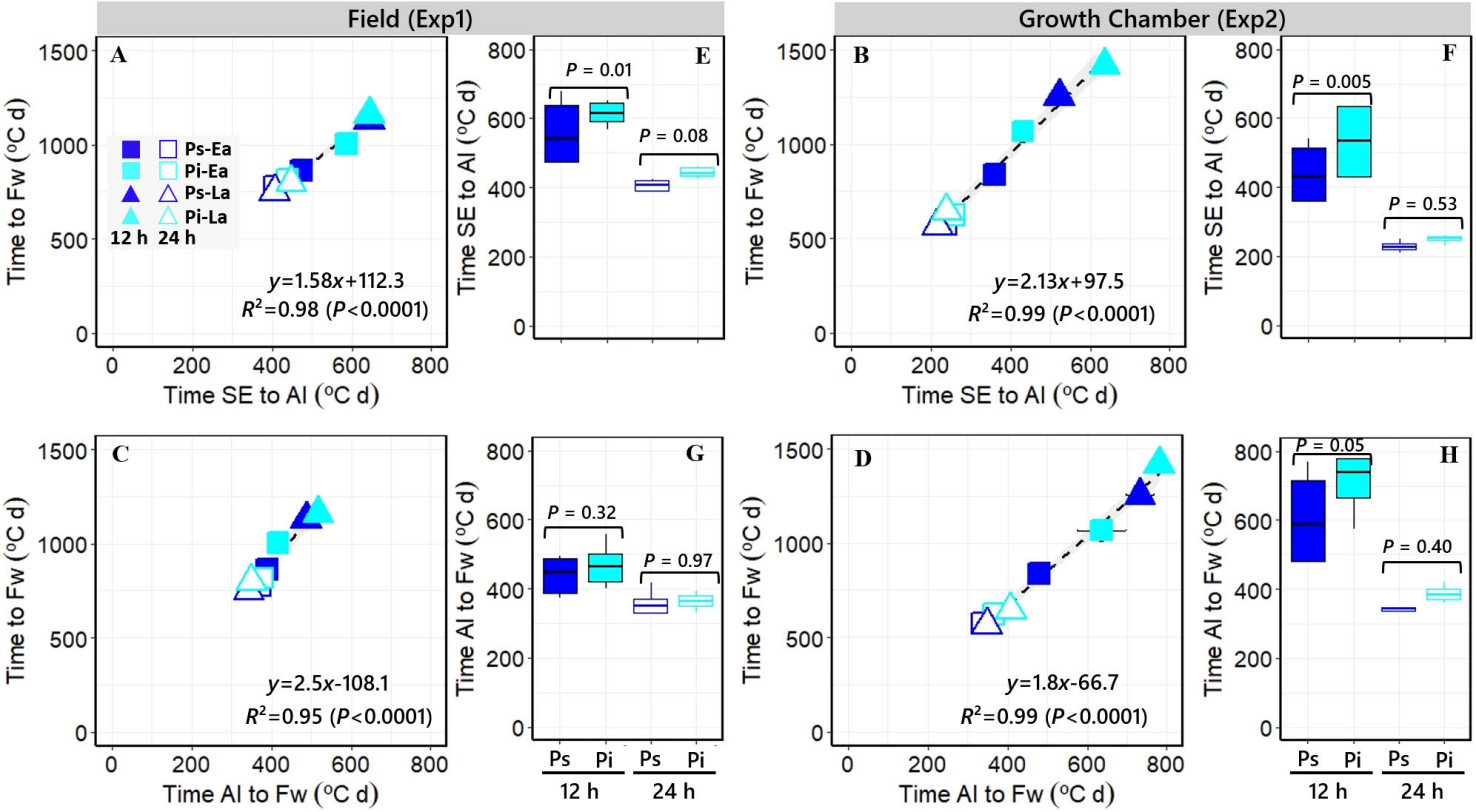
**(A–D)** Relationships between the durations of the whole phase from seedling emergence (SE) to flowering (Fw) and that of its component phases either from SE to awn initiation [AI; **(A, B)** in Exp1 and Exp2, respectively] or from AI to Fw [**(C, D)** in Exp1 and Exp2, respectively]. The segments on the symbols represent the standard errors of the means (not seen when smaller than the size of the symbol). Open and closed symbols correspond to long and short photoperiods, respectively. Squares: *PhyC-e*; triangles: *PhyC-l*; dark blue symbols: *Ppd-H1*; and light blue symbols: *ppd-H1*. **(E–H)** Boxplots grouping the NILs with *Ppd-H1*-sensitive (dark blue boxplots) and *ppd-H1*-insensitive (light blue boxplots) alleles across *PHYC* backgrounds for the duration of the phases from SE to AI [**(E, F)** in Exp1 and Exp2, respectively] and from AI to Fw [**(G, H)** in Exp1 and Exp2, respectively] under short (closed) and long (open) photoperiods, including the level of significance (*p*-value) of the difference between NILs with contrasting *PPD-H1* alleles within each photoperiod treatment. In the field experiment, a 12-h photoperiod corresponds to the average of the period from seedling emergence to flowering.

Across all sources of variation, time to Fw was very strongly related (*R* ^2^ > 0.95; *p* < 0.001) to the duration of both component phases, from SE to AI (**Figures 3A, B**) and from AI to Fw (**Figures 3C, D**) consistently across the two different experiments (i.e., the effect of all treatments together on time to Fw was due to effects on both phases). However, a major part of the similarly strong relationships of time to Fw with its two component phases was driven by the photoperiod growing condition: the phases of leaf and spikelet initiation and of floret development within spikelets (and then of spikelet survival) were both similarly affected by the photoperiodic condition in both experiments (**Figure 3**). Focusing on the effects produced by the *PPD-H1* alleles, the delay in Fw produced by the insensitivity allele was only significant under short photoperiod conditions in both experiments (see boxplots in **Figure 2**), and this effect was clearer in the duration of the period from SE to AI than in that from AI to Fw (although the latter also showed a consistent, though non-significant, trend to be delayed due to the action of the *ppd-H1* allele; open boxplots in **Figure 3**). Thus, under these relatively short photoperiods, there seemed to have been a sort of knock-on effect caused by the *ppd-H1* allele, clearly lengthening the duration of the SE-AI phase but also tending to lengthen that of the AI-Fw phase.

### 3.2 Dynamics of leaf appearance and tillering

**Figure 4:**
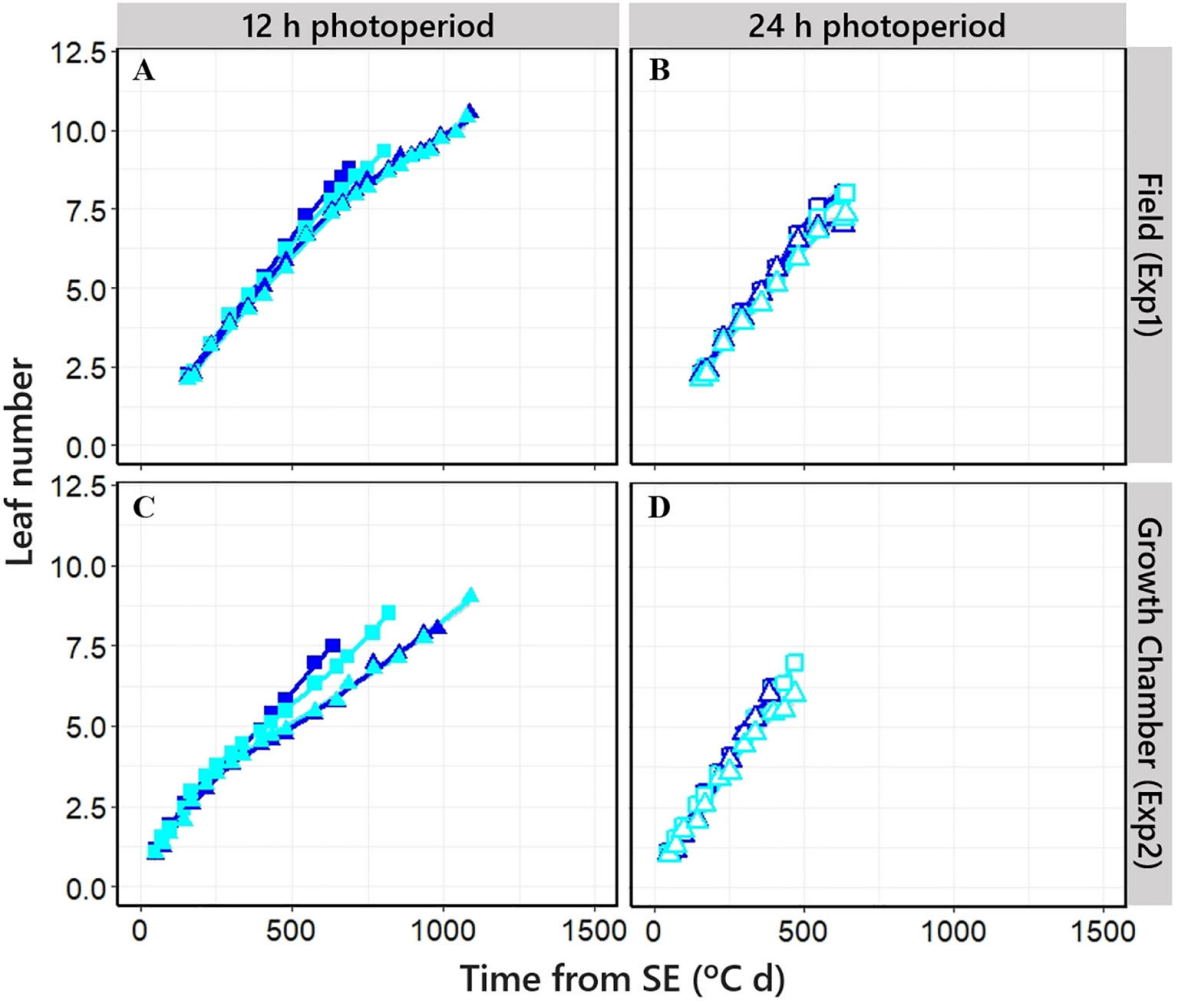
Relationship between cumulative leaf number on main shoot and time from seedling emergence in four different NILs grown at short [**(A, C)**; closed symbols] and long photoperiod [**B, D)**; open symbols] in Exp1 (top) and Exp2 (bottom). Square: *PhyC-e*; triangle: *PhyC-l*. Dark blue symbols: *Ppd-H1*; light blue symbols: *ppd-H1.* In the field experiment, a 12-h photoperiod corresponds to the average of the period from seedling emergence to flowering.

The leaf appearance rate was constant for the initial ca. six leaves across NILs, as indicated by the linear relationships when plotting leaf number vs. thermal time. However, when the final leaf number (FLN) was clearly higher than this threshold (particularly under short photoperiod), the rate of leaf appearance for the later leaves decreased, exhibiting a bilinear relationship between leaf number and thermal time across NILs (and the higher the FLN, the stronger the increase in phyllochron; **Figures 4A, C**).

**Figure 5:**
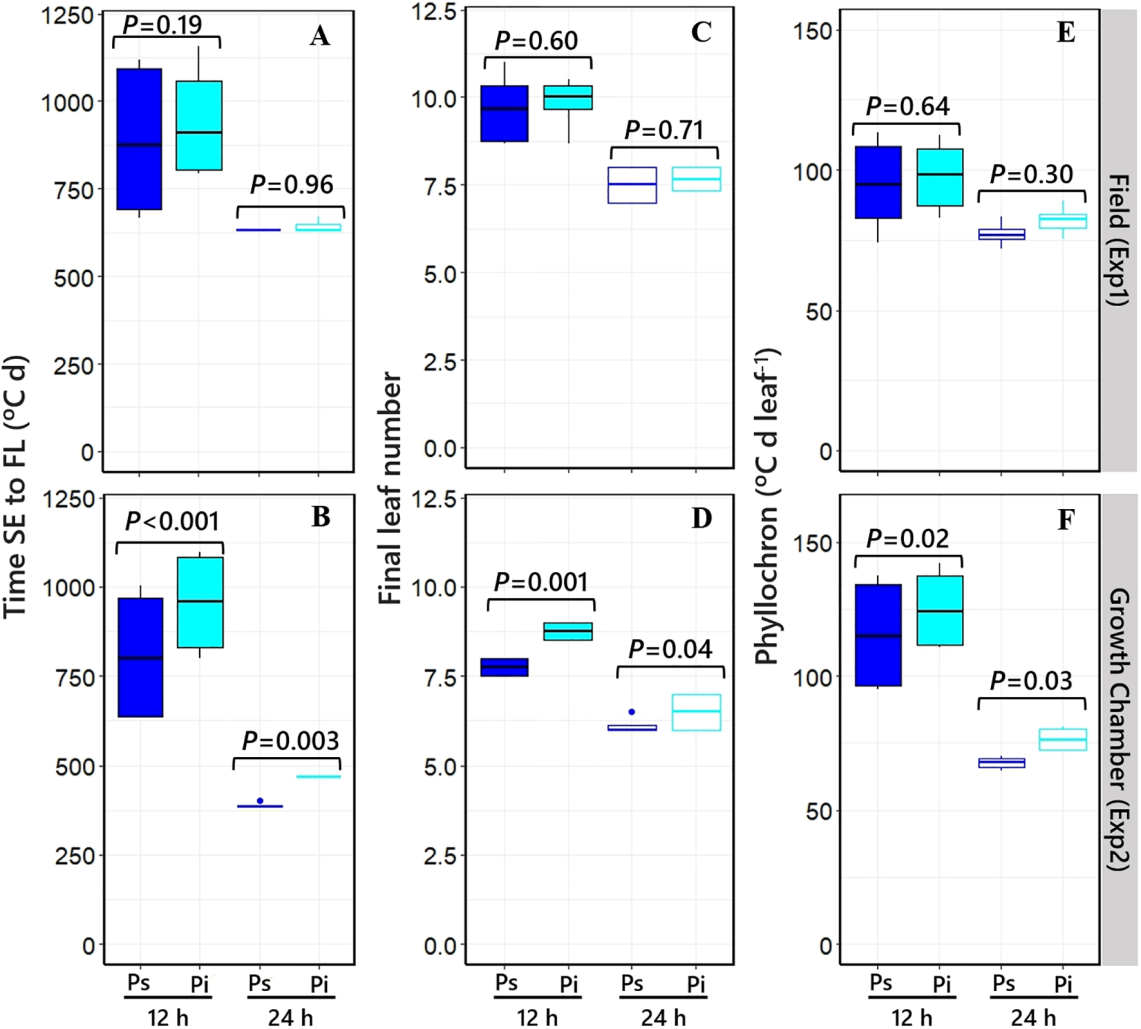
Boxplot grouping the NILs with *Ppd-H1*-sensitive (dark blue boxplots) and *ppd-H1*-insensitive (light blue boxplots) alleles across *PHYC* backgrounds for the duration of the phase from seedling emergence to flag leaf [**(A, B)** in Exp1 and Exp2, respectively], final leaf number [**(C, D)** in Exp1 and Exp2, respectively], and average phyllochron [**(E, F)** in Exp1 and Exp2, respectively] under short (closed) and long (open) photoperiods, including the level of significance (*p*-value) of the difference between NILs with contrasting *PPD-H1* alleles within each photoperiod treatment. In the field experiment, a 12-h photoperiod corresponds to the average of the period from seedling emergence to flowering.

Time to the appearance of the flag leaf was clearly affected by photoperiod across NILs in both experiments (*cf.* the pairs of boxplots under short and long photoperiods in **Figures 5A, B**), driven by the effects of the photoperiod condition on both FLN and average phyllochron (**Figures 5C, E** [Exp1], **Figures 5D, F** [Exp2]).

The allelic form of the *PPD-H1* gene affected phyllochron slightly but consistently across photoperiods and experiments, although the effect was significant only under controlled conditions (**Figures 5E, F**). Under long photoperiods, NILs having *Ppd-H1*-sensitive and *ppd-H1*-insensitive alleles had phyllochrons of, on average, 77°C and 82°C day^−1^ leaf in Exp1 and 68°C and 76°C day^−1^ leaf in Exp2, respectively (**Figures 5E, F**). Under a short photoperiod, NILs having the *Ppd-H1*-sensitive allele had on average a consistently shorter phyllochron (95°C and 112°C day^−1^ leaf) than those carrying the *ppd-H1*-insensitive allele (98°C and 125°C day^−1^ leaf, in Exp1 and Exp2, respectively). *PPD-H1* alleles did not affect FLN in Exp1 under either of the two photoperiod conditions (**Figure 5C**). However, in Exp2, NILs having *ppd-H1*-insensitive alleles increased FLN, though rather slightly by less than one leaf, in both photoperiod conditions (**Figure 5D**).

**Figure 6:**
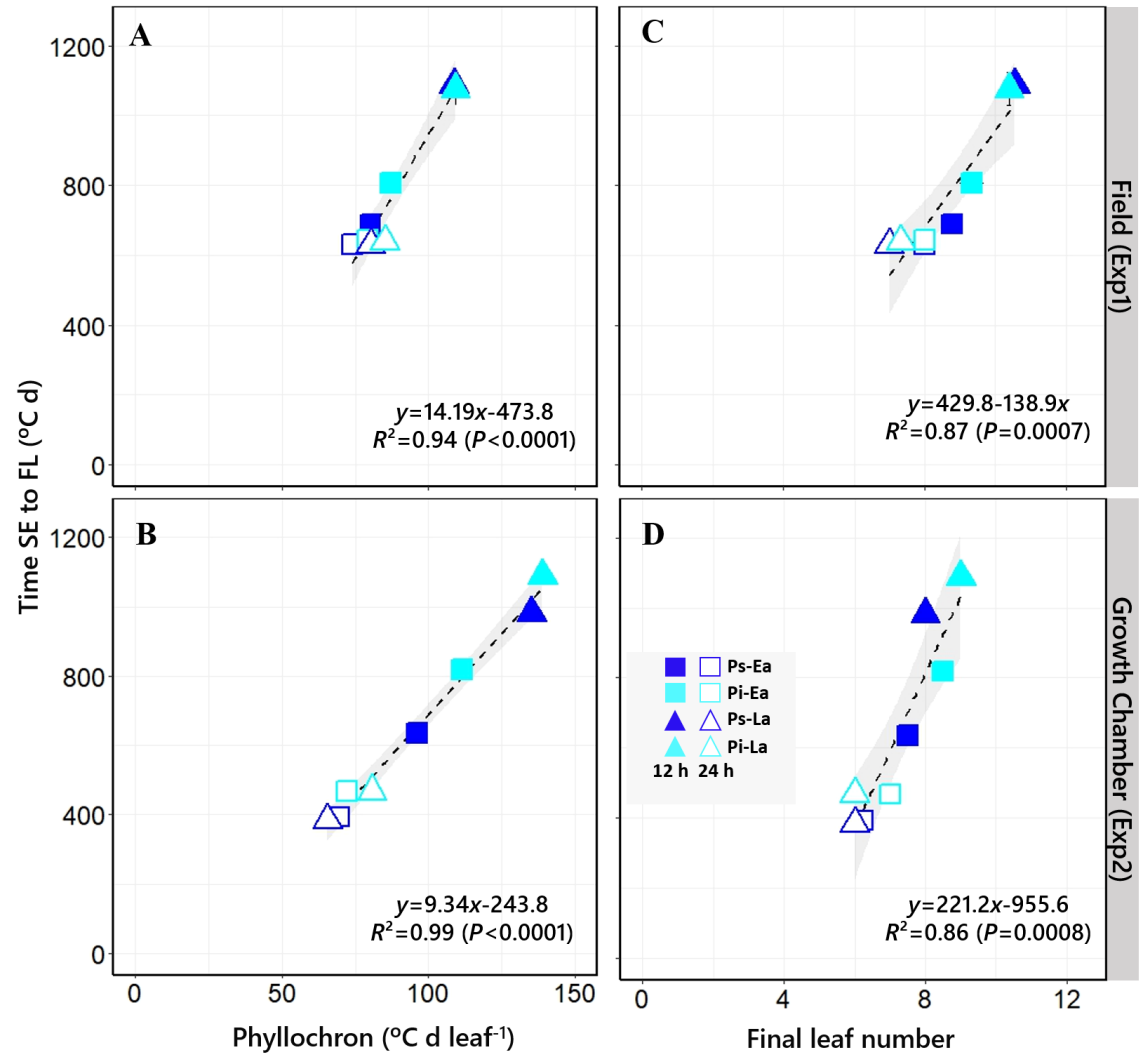
Relationship between the duration of the phase from seedling emergence to flag leaf and phyllochron [**(A, B)** in Exp1 and Exp2, respectively] or final leaf number [**(C, D)** in Exp1 and Exp2, respectively]. Bars on the symbols represent the standard errors of the means (not seen when smaller than the size of the symbol). The equation, coefficient of determination (*R* ^2^), and level of significance (*p*-value) of the linear regression are shown. Open and closed symbols correspond to long and short photoperiods, respectively. Square: *PhyC-e*; triangle: *PhyC-l*. Dark blue symbols: *Ppd-H1*; light blue symbols: *ppd-H1.* In the field experiment, a 12-h photoperiod corresponds to the average of the period from seedling emergence to flowering.

Thermal time to flag leaf was better explained by phyllochron (*R* ^2^ = 0.94 and *R* ^2^ = 0.99 for Exp1 and Exp2, respectively; **Figures 6A, B**) than by FLN (*R* ^2^ = 0.87 and *R* ^2^ = 0.86 for Exp1 and Exp2, respectively) (**Figures 6C, D**).

**Figure 7:**
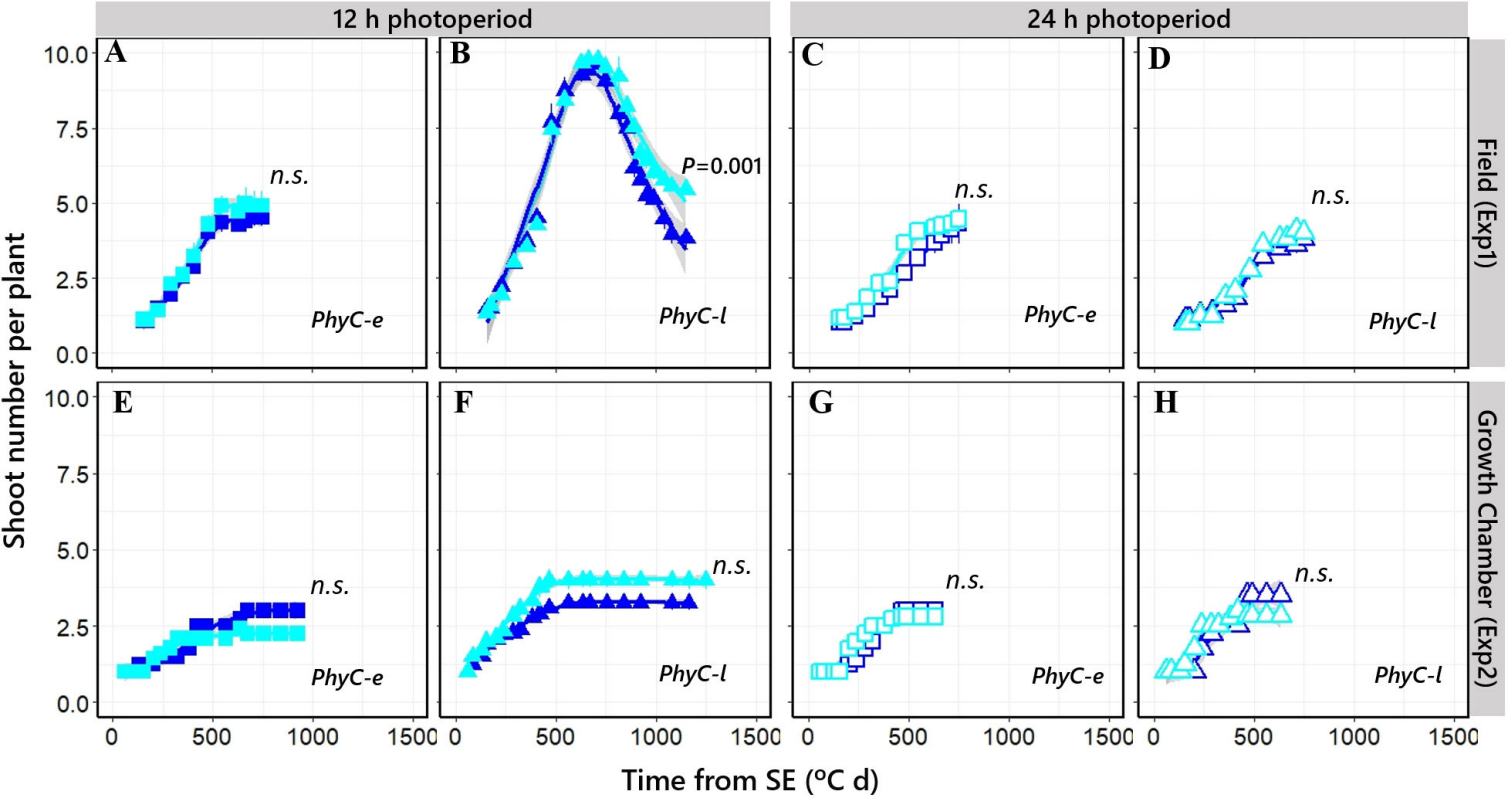
Relationship between shoot number per plant and time from seedling emergence in four different NILs grown at short photoperiod [closed symbols; **(A, B, E, F)**] and long photoperiod [open symbols; **(C, D, G, H)**] in Exp1 (top) and Exp2 (bottom). Square: *PhyC-e*; triangle: *PhyC-l*. Dark blue symbols: *Ppd-H1*; light blue symbols: *ppd-H1.* Bars on the symbols represent the standard errors of the means (not seen when smaller than the size of the symbol). In the field experiment, a 12-h photoperiod corresponds to the average of the period from seedling emergence to flowering. n.s., not significant.

Tillering dynamics was similar across experiments and NILs with relatively limited tillering and consequently having very little tiller mortality (**Figures 7A, C–E, G**, **H**). The effects of *PPD-H1* alleles were not large nor consistent for all cases, but when the environmental background was the short photoperiod and the genetic background included the *PhyC-l* allele, in general, the NIL with the insensitive *ppd-H1* allele produced more spikes per plant due to reduced tiller mortality in Exp1 (**Figure 7B**) and maintained tillering a bit longer in Exp2 (**Figure 7F**).

### 3.3 Apex development

In general, in the central spikelets of the main shoot spike, awn initiation and flag leaf stages coincided with floret developmental stages of W4.75 and W8, respectively, of the scale of Waddington et al. (1983), varying only very slightly across NILs, experiments, and photoperiod conditions (**Supplementary Figure S1**).

**Figure 8:**
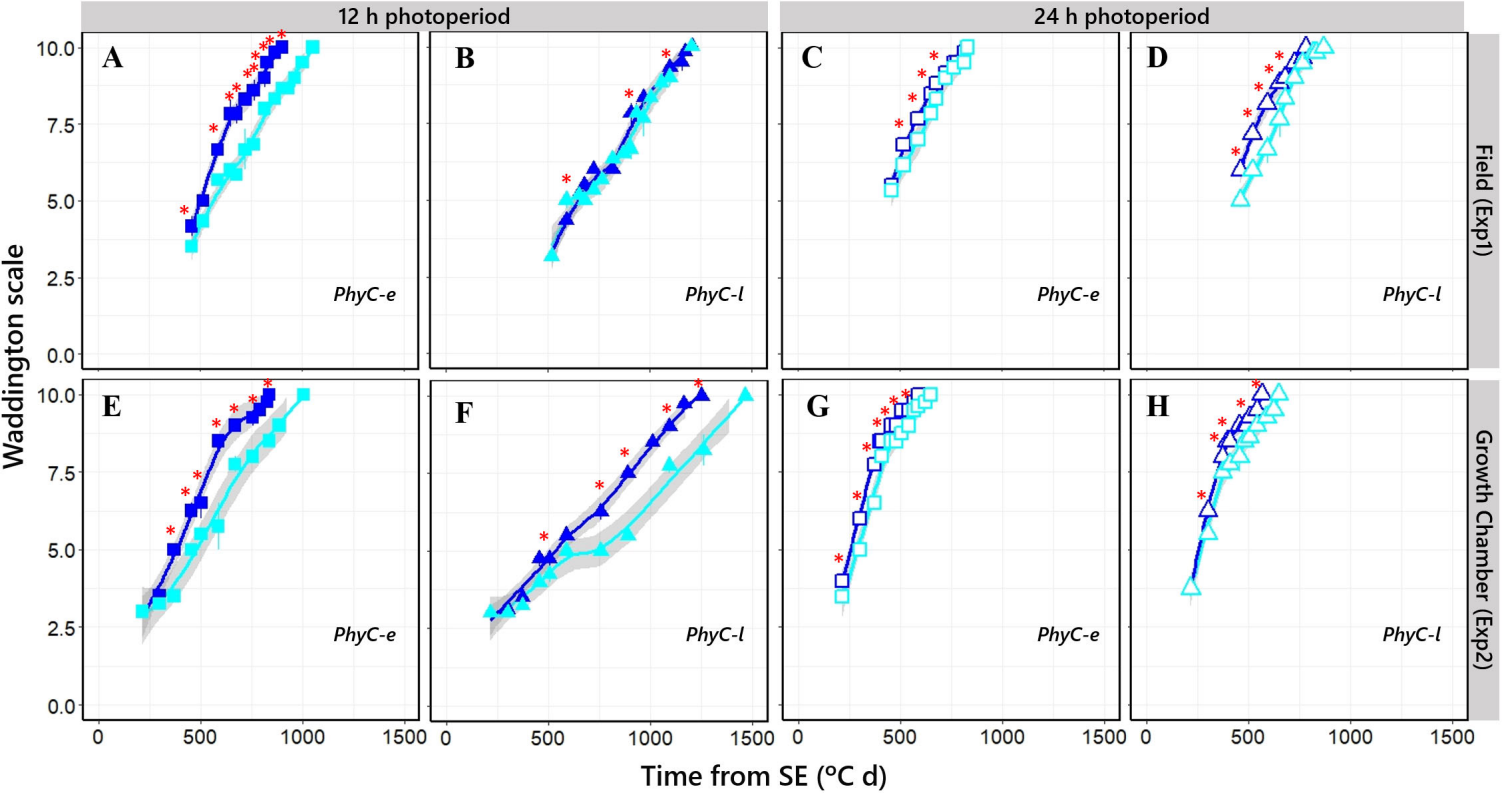
Floret development stages, assessed by the Waddington scale, along time from seedling emergence under short [closed symbols; **(A, B, E, F)**] and long [open symbols; **(C, D, G, H)**] photoperiod conditions in *Ppd-H1*-sensitive (dark blue symbols) and *ppd-H1*-insensitive (light blue symbols) alleles. Top, Exp1; bottom, Exp2. Values at each timing are means of three (Exp1) or two (Exp2) plants. Significant differences in floret development stage at each particular timing of sampling are indicated by asterisks (^*^ *p* < 0.05).

NILs having sensitive *Ppd-H1* alleles slightly accelerated flowering by promoting early shoot apex development, whose magnitude depended on the photoperiod condition and *PHYC* genetic background (**Figure 8**; **Supplementary Figures S2**, **S3**). This effect of *PPD-H1* was only slight on long days (**Figures 8C, D, G, H**), when the time to flowering was not significantly delayed (see above). Under short photoperiod conditions, florets in the insensitive *ppd-H1* lines showed a much clearer development deceleration (**Figures 8A, E, F**), except for the *PhyC-l* background under natural photoperiod in Exp1, where the difference was not significant (**Figure 8B**).

This effect of *PPD-H1* is reflected in the developmental rates of a particular organ (florets), which has been observed for the phenological effects on flowering time.

## 4 Discussion

The effects of *PPD-H1* alleles on the components of time to flowering could be assessed both in terms of subphase durations (Slafer and Rawson, 1994; Kirby et al., 1999) and in terms of the number of leaves initiated and phyllochron (Slafer and Rawson, 1997; Jamieson et al., 1998). Many studies showed that under long days of 16–18 h, the time to flowering was significantly delayed by the action of the insensitivity allele *ppd-H1* (Laurie et al., 1994; Turner et al., 2005; Digel et al., 2015; Parrado et al., 2023; Slafer et al., 2023a). Indeed, introgressing this allele was critical for spring barley production at high latitudes to avoid the extremely short cycle of the sensitive cultivars possessing the *Ppd-H1* allele (Fernández-Calleja et al., 2021 and references quoted therein). However, lengthening the cycle under long days by introgressing insensitivity to photoperiod would be counterintuitive for a long-day plant (Slafer et al., 2009), and therefore, at photoperiods even longer than 21 h, lines should flower similarly (Parrado et al., 2023). We found here that when plants were grown at a 24-h photoperiod, the effect of *ppd-H1* allele on phenology was negligible, which is consistent with a recent study where we uncovered responses of barley lines with contrasting photoperiod sensitivity to extreme photoperiods (Parrado et al., 2023).

In our set of NILs, time to flowering was related to the duration of both the vegetative plus the early reproductive phase (i.e., from SE to AI) and the late reproductive phase, mainly driven by variability in the short photoperiod treatment generated by the *PHYC* alleles, which is consistent with the findings of Pankin et al. (2014). Even though there was a relationship between the duration of the two phases, as reported in other studies (Appleyard et al., 1982; González et al., 2005; Whitechurch et al., 2007b; Borràs-Gelonch et al., 2012), the idea that the duration of these phases may be independent is still valid. This is evident when screening a large number of genotypes (e.g., Kitchen and Rasmusson, 1983; Kernich et al., 1997; Whitechurch et al., 2007a; Borràs et al., 2009), but the independent duration of these phases would be controlled by other minor genes (Borràs-Gelonch et al., 2010; Alqudah et al., 2014), as the major developmental genes like *PPD-H1* seem to affect all preflowering phases, in line with what previously reported in barley (Borràs-Gelonch et al., 2012; Digel et al., 2015; Fernández-Calleja et al., 2021), as well as in wheat (González et al., 2005).

Although there were slight phenological variations within contrasting photoperiods, the SE-AI period was more affected by the *ppd-H1* allele than the AI-Fw period. However, studies conducted under long photoperiods of 16 h showed that *ppd-H1* delayed both early and late reproductive development (Digel et al., 2015; Ejaz and von Korff, 2017). This would suggest that preflowering phases may vary in their sensitivity to *PPD-H1* depending on the duration of the day. Furthermore, it is well known that the impact of *PPD-H1* on time to flowering may be influenced by genetic background (Laurie et al., 1995; Szucs et al., 2006; Hemming et al., 2008; Nishida et al., 2013; Turner et al., 2013; Pankin et al., 2014). Therefore, since AI-Fw was significantly influenced by the photoperiodic environment (12 h vs. 24 h) and *PPD-H1* had a negligible effect on this period within the photoperiod treatments, the duration of AI-Fw must be regulated by another photoperiod response gene (or potentially interacting with *PPD-H1*) that has not yet been identified.

An overall view (including photoperiod treatment and *PHYC* background) of relationships between the number of leaves initiated, rate of leaf appearance, and time to flowering would suggest that most of the effects of *PPD-H1* alleles on time to flowering can be seen as a consequence of the effects on both FLN and phyllochron, as reported when the treatments were not particular photoperiod-sensitivity genes by Kirby (1990) and Miralles et al. (2021) or specifically *PPD-H1* alleles exposed to different photoperiods (12 h vs. 16 h) during the early phase of development (Digel et al., 2015). The increase in phyllochron observed under short days can be attributed to a significant decrease in the rate of leaf appearance after the first six leaves had appeared, as previously documented for wheat by Slafer and Rawson (1997), leading to the lengthening of the AI-Fw stage, complementing the most relevant effect of this gene on the duration of the phases of leaf and spikelet development. The interplay between FLN and phyllochron ends up making *PPD-H1* affect both phases of time to Fw (i.e., through reducing the rate of development in the vegetative phase, the insensitive allele increases FLN, and then as the last leaves appear more slowly than the first leaves, this generates a carry-over effect on the duration of the late reproductive phase). This finding is consistent with that showing that the phyllochron of the initial leaves was unaffected by the *PPD-1* alleles in wheat, while that of the later leaves was sensitive (González et al., 2005). However, evaluating the effect of *PPD-H1* within photoperiodic environments and *PHYC* backgrounds, the elongation of the SE-AI period induced by the *ppd-H1* allele could be due to an elongation of the early reproductive phase and not of the vegetative stage, as suggested by a negligible change in the final number of leaves between NILs. This is consistent with previous work where *PPD-H1* did not induce changes in the vegetative stage (Pankin et al., 2014; Digel et al., 2015; Ejaz and von Korff, 2017).

Although it has a slight effect on the duration of the AI-Fw period within photoperiod treatment, *PPD-H1* seems to have affected floret development. This is, in turn, commensurate with the deceleration of floret primordia development under shorter photoperiods found in the present study, in line with what had been suggested by Digel et al. (2015).

As *PPD-H1* alleles did not affect the number of tillers and their dynamics (there was just a trend with *PHYC-l* under a short photoperiod), any effect of this gene on yield components will be mainly driven by an effect on spike fertility rather than by the number of spikes per unit land area, at least at the agronomically sound sowing densities used here.

## Data Availability

The raw data supporting the conclusions of this article will be made available by the authors, without undue reservation.

## Footnotes

1 Although floral initiation does normally occur earlier, with the first spikelets initiated as single ridges (Delécolle et al., 1989; Kirby, 1990; Ochagavía et al., 2018), it has been traditionally assumed that the stage of double ridge shows the transition from vegetative to reproductive apex (Slafer et al., 2021) because it is the first microscopic evidence that the apex is indubitably reproductive.

2 Also referred to in the literature as the mutant *ppd-H1* allele, inducing reduced photoperiod sensitivity (Turner et al., 2005; Ejaz and von Korff, 2017).

3 Due to the dedicated system used to install the plots in Exp1 and the selection of uniform plants after vernalising them in Exp2, each of the plants was very representative.
