# Supplementary material for: Dynamics of apex and leaf development in barley as affected by PPD-H1 alleles in two contrasting PHYC backgrounds under short or long photoperiod
Source: Front Plant Sci. 2024 Sep 3;15:1398698. doi: 10.3389/fpls.2024.1398698 (PMC11405203; doi:10.3389/fpls.2024.1398698)
Supplement: Supplementary file 1 [file DataSheet1.docx]

Dynamics of apex and leaf development in barley as affected by PPD-H1 alleles in two contrasting PHYC backgrounds under short or long photoperiod

Jorge D. Parrado, Roxana Savin, Gustavo A. Slafer

Supplementary Tables and Figures

1. Supplementary Tables

Table S1: Mean squares from the analyses of variance for the main developmental traits in field experiment (Exp1).

| **Source of variation** | **df** | **Fw** | **SE-AI** | **AI-Fw** | **SE-FL** | **FLN** | **Phyllochron** |
| --- | --- | --- | --- | --- | --- | --- | --- |
|  |  | **(ºC d)** | **(ºC d)** | **(ºC d)** | **(ºC d)** | **(#leaves)** | **(ºC d)** |
| **Block** | 2 | 402ns.. | 840*… | 80ns.... | 3,602ns.. | 0.1ns.. | 43ns.. |
| **Photoperiod** | 1 | 390,533**. | 158,909*** | 51,208*... | 466,911**. | 28.5**.. | 1,652*-.- |
| **Block*photoperiod (Random)** | 2 | 1,234ns.. | 44ns…. | 1,638ns.. | 2,840ns.. | 0.1ns-- | 18ns-- |
| **Genotype** | 3 | 27,185*** | 12,721*** | 3,040*… | 59,498*** | 0.3*--- | 545*** |
| **Photoperiod*Genotype** | 3 | 30,740*** | 8,046*** | 8,359*** | 59,389*** | 2.7*** | 196*** |

Fw: flowering; SE-AI: seedling emergence to awn initiation; AI-Fw: awn initiation to flowering; SE-FL: seedling emergence to flag leaf; FLN: final leaf numbers. ns, *, **, ***, indicates non-significant, and significance at the 0.05, 0.01, and 0.001 probability level, respectively.

Table S2: Mean squares from the analyses of variance for the main developmental traits in growth chamber experiment (Exp2).

| **Source of variation** | **df** | **Fw** | **SE-AI** | **AI-Fw** | **SE-FL** | **FLN** | **Phyllochron** |
| --- | --- | --- | --- | --- | --- | --- | --- |
|  |  | **(ºC d)** | **(ºC d)** | **(ºC d)** | **(ºC d)** | **(#leaves)** | **(ºC d)** |
| **Photoperiod** | 1 | 9,364*** | 248,004*** | 342,225*** | 820,836*** | 15***….. | 820,836*** |
| ***PPD-H1*** | 1 | 341*** | 12,996*** | 21,609**.. | 50,625*** | 1.9***... | 50,625*** |
| ***PHYC*** | 1 | 1,276*** | 26,244*** | 50,625*** | 91,809*** | 0.0ns…. | 91,809*** |
| **Photoperiod**PPD-H1*** | 1 | 0.6ns | 5,184*** | 3,249ns-- | 4,356**.. | 0.4**..... | 4,356**.. |
| **Photoperiod**PHYC*** | 1 | 959*** | 41,616*** | 29,241**.. | 97,344*** | 1.3***... | 97,344*** |
| ***PPD-H1***PHYC*** | 1 | 0.2ns | 576ns... | 1,521ns.. | 1,089ns.. | 0.1*…... | 1,089ns.. |
| **Photoperiod**PPD-H1*PHYC*** | 1 | 148*** | 324ns... | 4,761ns.. | 1,764*… | 0.1*…... | 1,764*… |

Fw: flowering; SE-AI: seedling emergence to awn initiation; AI-Fw: awn initiation to flowering; SE-FL: seedling emergence to flag leaf; FLN: final leaf numbers. ns, *, **, ***, indicates non-significant, and significance at the 0.05, 0.01, and 0.001 probability level, respectively.

2. Supplementary Figures


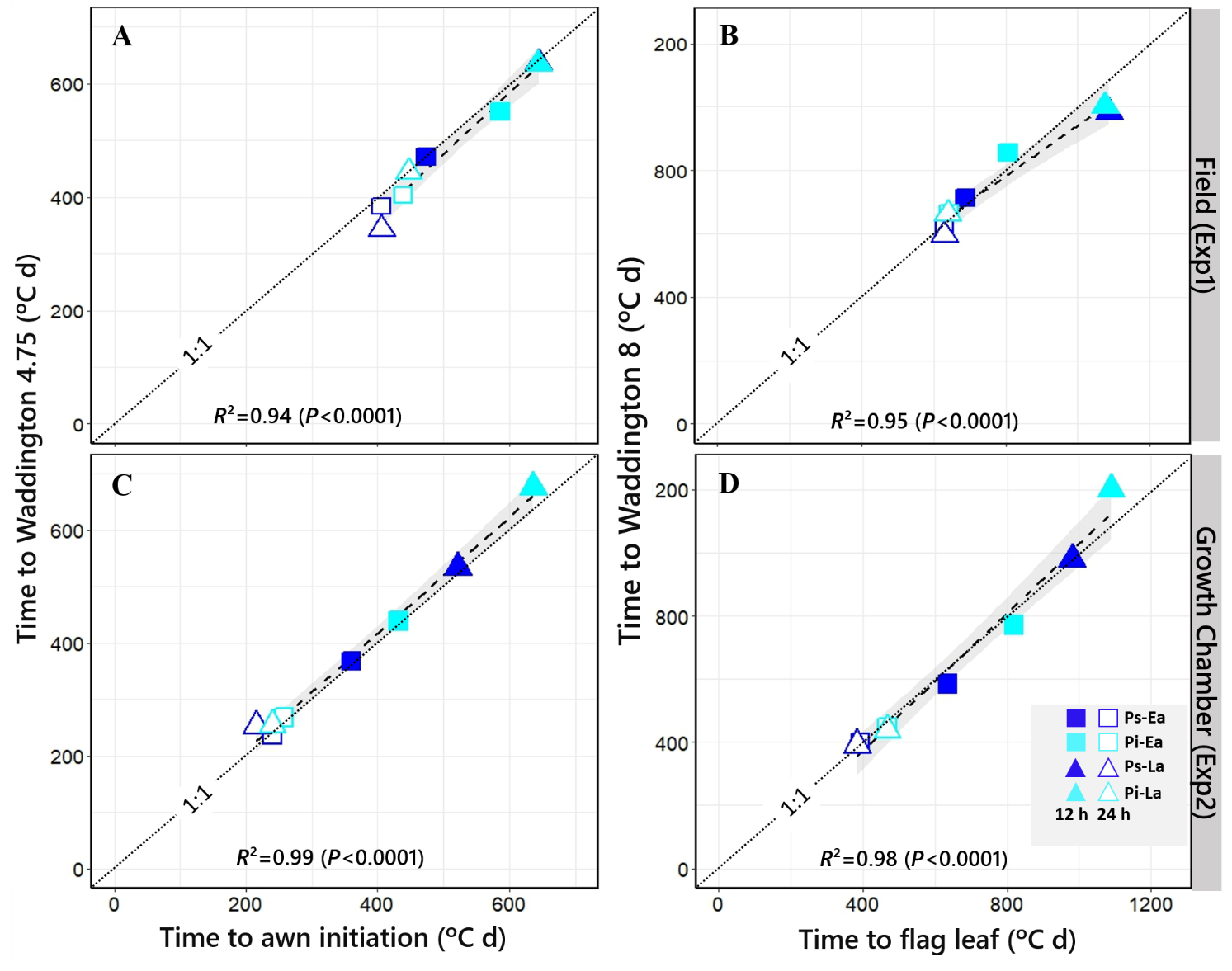


**Supplementary Figure 1.** Leaf panels: relationship between the duration of the phase from seedling emergence to Waddington 4.75 stage and to awn initiation (A and C in Exp1 and Exp2, respectively). Right panels: relationship between the duration of the phase from seedling emergence to Waddington 8 stage and to flag leaf: Z39 (B and D in Exp1 and Exp2, respectively). Waddington data correspond to the fitting of the dynamics of floret development during the whole period SE-Fw in the central position of the rachis of main shoot spikes. Open and closed symbols correspond to long and natural/short photoperiod, respectively. Square: *PhyC-e*; triangle: *PhyC-l*. Dark blue symbols: *Ppd-H1*; Light blue symbols: *ppd-H1.*

.
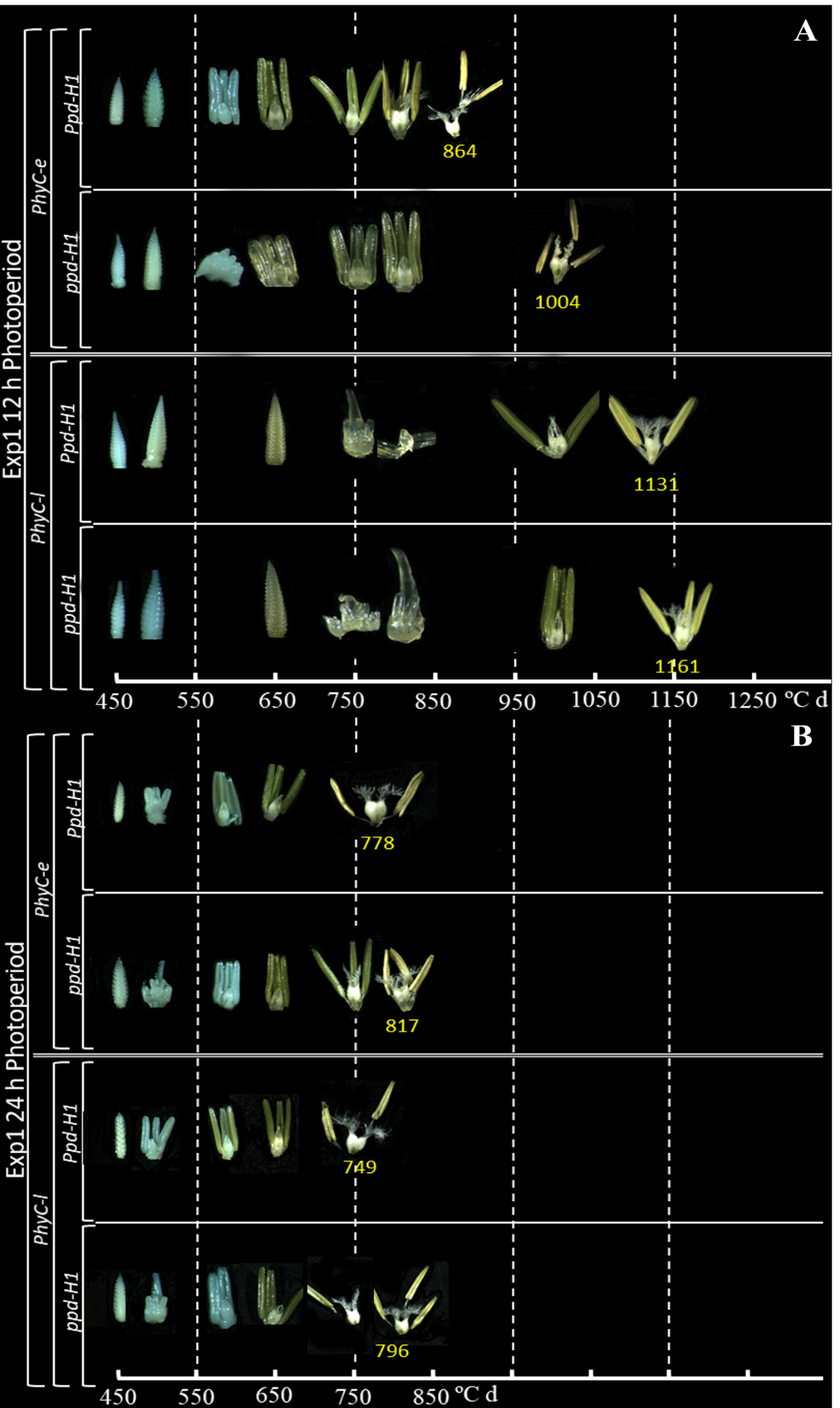


**Supplementary Figure 2.** A, B. Images of apex/floret development taken under microscopy along time from seedling emergence to flowering in the field experiment. The natural photoperiod in this experiment had an average from seedling emergence to flowering of 12 h and the extended photoperiod was a constant 24 h (with lights in the field complementing natural photoperiod).


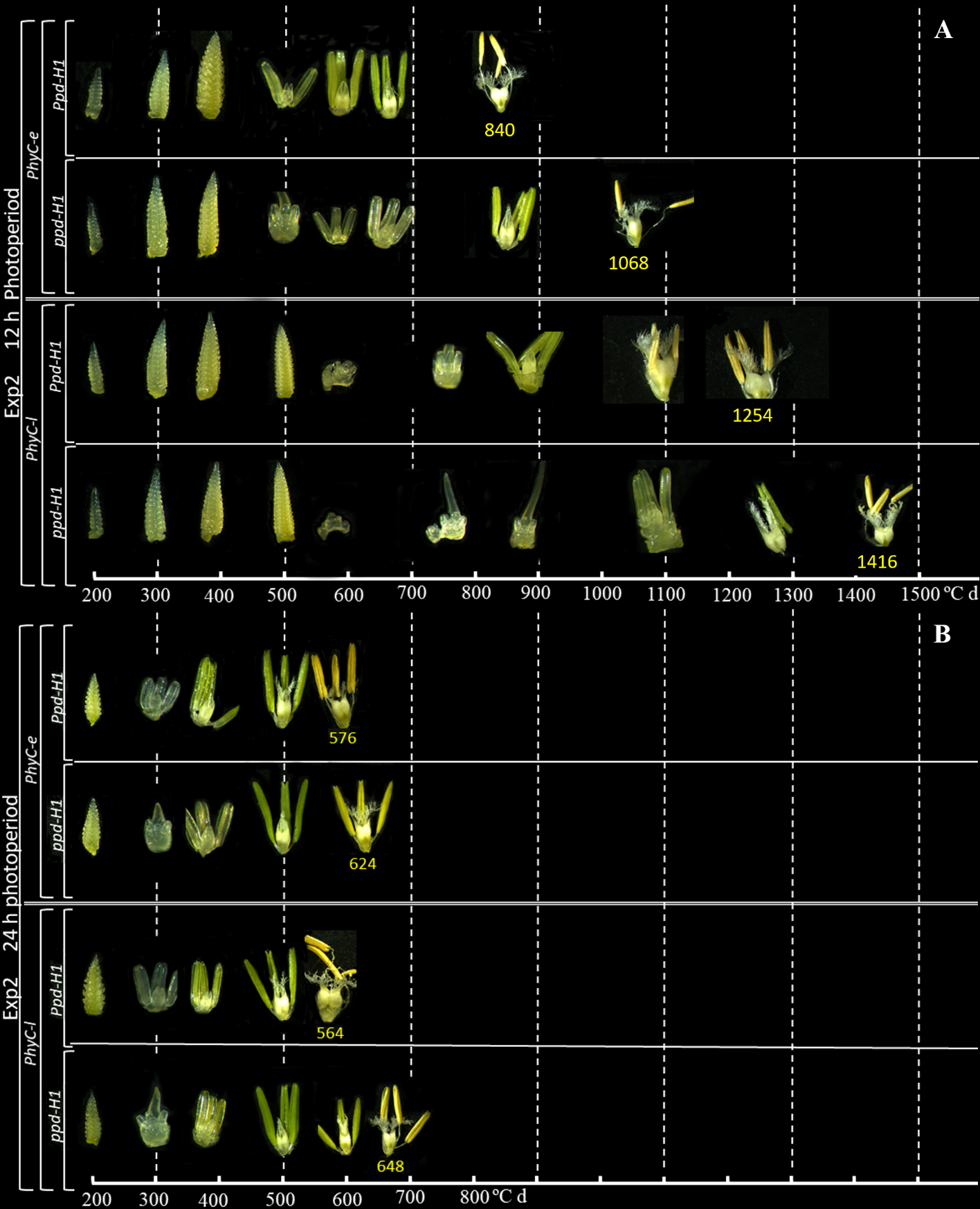


**Supplementary Figure 3.** Figure S3 A, B. Images of apex/floret development taken under microscopy along time from seedling emergence to flowering in the growth chamber experiment.
